# The isolation and mapping of a novel hydroxycinnamoyltransferase in the globe artichoke chlorogenic acid pathway

**DOI:** 10.1186/1471-2229-9-30

**Published:** 2009-03-18

**Authors:** Cinzia Comino, Alain Hehn, Andrea Moglia, Barbara Menin, Frédéric Bourgaud, Sergio Lanteri, Ezio Portis

**Affiliations:** 1 DiVaPRA Plant Genetics and Breeding, University of Torino 10095, Grugliasco (Torino), Italy; 2 UMR 1121 Nancy Université (INPL)-INRA, Agronomie Environnement Nancy-Colmar 2 avenue de la Forêt de Haye 54505 Vandoeuvre-lès-Nancy, France

## Abstract

**Background:**

The leaves of globe artichoke and cultivated cardoon (*Cynara cardunculus *L.) have significant pharmaceutical properties, which mainly result from their high content of polyphenolic compounds such as monocaffeoylquinic and dicaffeoylquinic acid (DCQ), and a range of flavonoid compounds.

**Results:**

Hydroxycinnamoyl-CoA:quinate hydroxycinnamoyltransferase (HQT) encoding genes have been isolated from both globe artichoke and cultivated cardoon (GenBank accessions DQ915589 and DQ915590, respectively) using CODEHOP and PCR-RACE. A phylogenetic analysis revealed that their sequences belong to one of the major acyltransferase groups (anthranilate N-hydroxycinnamoyl/benzoyltransferase). The heterologous expression of globe artichoke HQT in *E. coli *showed that this enzyme can catalyze the esterification of quinic acid with caffeoyl-CoA or *p*-coumaroyl-CoA to generate, respectively, chlorogenic acid (CGA) and *p*-coumaroyl quinate. Real time PCR experiments demonstrated an increase in the expression level of HQT in UV-C treated leaves, and established a correlation between the synthesis of phenolic acids and protection against damage due to abiotic stress. The HQT gene, together with a gene encoding hydroxycinnamoyl-CoA:shikimate/quinate hydroxycinnamoyltransferase (HCT) previously isolated from globe artichoke, have been incorporated within the developing globe artichoke linkage maps.

**Conclusion:**

A novel acyltransferase involved in the biosynthesis of CGA in globe artichoke has been isolated, characterized and mapped. This is a good basis for our effort to understand the genetic basis of phenylpropanoid (PP) biosynthesis in *C. cardunculus*.

## Background

*Cynara cardunculus *L. (2n = 2x = 34) is an allogamous species native to the Mediterranean basin, belonging to the family Asteraceae, order Asterales. The species includes three subspecies: the globe artichoke (var. *scolymus *L.), which is grown for its edible immature inflorescence; the cultivated cardoon (var. *altilis *DC.), which produces fleshy stalks; and their common ancestor, the wild cardoon (var. *sylvestris *(Lamk) Fiori) [1-3]. Leaf extracts contain molecules of some pharmaceutical interest, including antibacterial [4-7] antioxidative [8,9] anti-HIV [10-12], hepatoprotective, choleretic [13], cholesterol biosynthesis inhibitory [14,15] and anticancer [16] activities. Many of these properties rely on specific phenylpropanoids (PPs), particularly 5-caffeoylquinic acid (chlorogenic acid, CGA) and di-caffeoylquinic (DCQ) acids, along with various flavonoid compounds [17,18]. The level and composition of the PP pool can vary considerably between organisms, tissues, developmental stages and in response to environmental conditions [19,20]. PP metabolism is induced by biotic and abiotic stresses such as wounding, UV-irradiation and pathogen attack [21,22]. Recently, Moglia *et al*. [23] have established that UV-C radiation enhances the level of caffeoylquinic acid in the globe artichoke.

**Figure 1:**
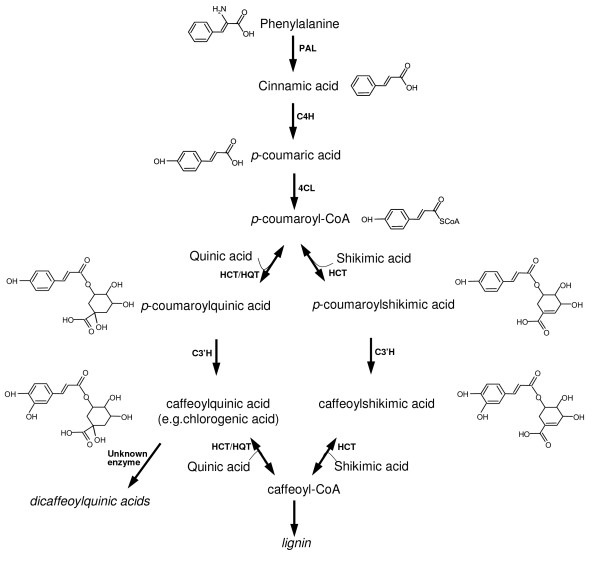
**A simplified diagram of enzymes and major products in the synthesis of chlorogenic acid in plants**. The product names appear between the arrows. Enzymes involved in this pathway are: PAL, phenylalanine ammonia lyase; C4H, cinnamate 4-hydroxylase; 4CL, 4-hydroxycinnamoyl-CoA ligase; HCT, hydroxycinnamoyl-CoA shikimate/quinate hydroxycinnamoyl transferase; HQT, hydroxycinnamoyl CoA quinate hydroxycinnamoyl transferase; C3'H, *p*-coumaroyl ester 3'-hydroxylase.

The CGA biosynthesis pathway has been the target of some detailed research, mainly focused among Solanaceae species [24-26] (Fig. 1). Even though little direct information is as yet available concerning the biosynthesis of di- and tri-caffeoylquinic acid, the prior accumulation of CGA does appear to be necessary. Three distinct pathways have been proposed for the synthesis of CGA: (1) the trans-esterification of caffeoyl-CoA and quinic acid via hydroxycinnamoyl-CoA:quinate hydroxycinnamoyl transferase (HQT) activity [27,28]; (2) the hydroxylation of *p-*coumaroyl quinate to CGA [25]; and (3) the hydroxylation of *p-*coumaroyl shikimate to caffeoyl shikimic acid, which is then converted to caffeoyl-CoA, a substrate of hydroxycinnamoyl-CoA:shikimate hydroxycinnamoyl transferase HCT [24]. The silencing of the HQT gene in tobacco and tomato results in a 98% reduction in CGA level, but does not affect lignin formation, so in these species at least, the first two of these routes are probably responsible for the biosynthesis and accumulation of CGA [25]. On the other hand, a lowered HCT expression in tobacco [29], *Pinus radiata *[30] and *Medicago sativa *[31] changes lignin amount and composition, thereby implicating the third pathway in lignin biosynthesis. A fourth route, which uses caffeoyl-glucoside as the active intermediate, has been described in sweet potato [26]. Although the globe artichoke HCT sequence is similar to that of tobacco HCT, its activity is more closely related to that of tobacco and tomato HQT, in showing a preference for quinic over shikimic acid as acceptor [32].

Linkage maps, created for genes in biosynthetic pathways in several species, can be used to locate known genes of a pathway within a specific genomic region. [33,34]. The presence of allelic variation at the sequence level in genes of known biochemical functional is useful for candidate gene approaches [35]. Genetic maps of globe artichoke [36] have been based on observed segregation behaviour in an F_1 _population formed by the intercrossing of the two contrasting varieties 'Romanesco C3' (a late-maturing, non-spiny type) and 'Spinoso di Palermo' (an early-maturing spiny type).

Here, we report the isolation of the cDNA of a novel acyltransferase involved in *C. cardunculus *PP biosynthesis, and assess its leaf expression level as induced by UV-C irradiation. We also derive the map location of this gene, along with that of the HCT gene described by Comino *et al*. [32].

## Results

### Isolation and cloning of a full length HQT cDNA of globe artichoke and cardoon

**Figure 2:**
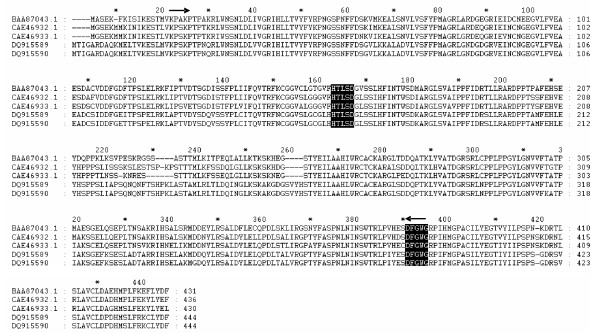
**Sequence alignment of HQT sequences belonging to the plant hydroxycinnamoyl transferase family**. BAA87043 from *Ipomoea batatas*; CAE46932 from *Nicotiana tabacum*; CAE46933 from *Lycopersicum esculentum*; DQ915589 (this work) from *Cynara cardunculus *var. *scolymus *and DQ915590 (this work) from *Cynara. cardunculus *var. *altilis*. Black boxes indicate structural motifs conserved in the acyltransferase family. The position of the primers designed with CODEHOP strategy is indicated by arrows.

**Figure 3:**
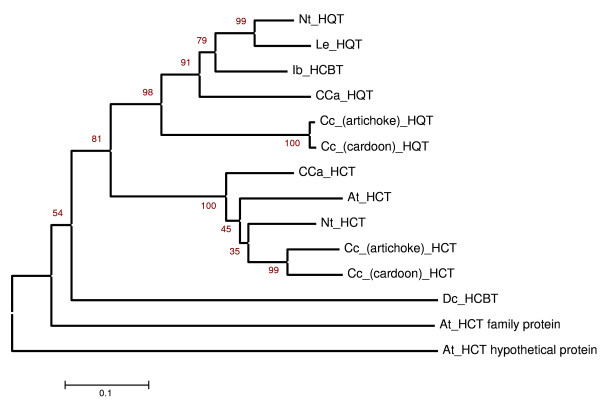
**Phylogenetic analysis of acyltransferases**. The tree was constructed by the neighbour-joining method with 10000 bootstrap replicates. The length of the lines indicates the relative distances between nodes. Protein sequences used for the alignment are: Dc_HCBT, anthranilate N-hydroxycinnamoyl/benzoyltransferase from *Dianthus caryophyllus *(CAB06427); Ib_HCBT, N-hydroxycinnamoyl/benzoyltransferase from *Ipomoea batatas *(BAA87043); At_HCT, shikimate/quinate hydroxycinnamoyltransferase from *Arabidopsis. thaliana *(ABH04595); Nt_HCT, shikimate/quinate hydroxycinnamoyltransferase from *Nicotiana tabacum *(CAD47830); Nt_HQT, hydroxycinnamoyl CoA quinate transferase from *Nicotiana tabacum *(CAE46932); Le_HQT, hydroxycinnamoyl CoA quinate transferase from *Lycopersicum esculentum *(CAE46933); Cca_HQT, hydroxycinnamoyl CoA quinate transferase from *Coffea canephora *(ABO77956); Cca_HCT, shikimate/quinate hydroxycinnamoyltransferase from *Coffea canephora *(ABO47805); NP_179497 and NP_200592, *Arabidopsis thaliana *genes encoding putative acyltransferases; Cc_(artichoke)_HCT, hydroxycinnamoyl CoA quinate transferase from *Cynara cardunculus *var. *scolymus *(AAZ80046); Cc_(cardoon)_HCT, hydroxycinnamoyl CoA quinate transferase from *Cynara cardunculus *var. *altilis *(AAZ80047); Cc_(artichoke)_HQT, quinate hydroxycinnamoyltransferase from *Cynara cardunculus *var. *scolymus *(ABK79689, this work) and Cc_(cardoon)_HQT, quinate hydroxycinnamoyltransferase from *Cynara cardunculus *var. *altilis *(ABK79690, this work).

CODEHOP [37] was used to target conserved acyltransferases in globe artichoke (see arrows in Fig. 2), resulting in the amplification of an incomplete internal acyltransferase-like sequence, which was extended in both globe artichoke and cultivated cardoon via a RACE strategy. Full-length cDNA sequences have been deposited in Genbank (DQ915589, DQ915590). The genes are of identical length (1335 bp) and their translation product is a 444 residue peptide with a molecular mass of ~50 kDa. The best matches obtained from a local alignment search within a non-redundant protein database (blastp) were with a sweet potato HCBT (70% identity, 85% similarity), and a tobacco HQT (72% identity, 84% similarity), which belongs to a multifunctional superfamily of plant acyltransferases [38]. The sequences contain a HTLSD peptide (aa 163–168, black boxes in Fig. 2), as does the HCT isolated by Comino *et al*. [32], matching the highly conserved HXXXD motif characteristic for acyl transfer proteins. The DFGWG block [38,39] present in other acyltransferases of the BAHD family [40] is present from aa 391 to 395 (Fig. 2, black boxes). Phylogenetic analyses confirmed that the isolated sequence showed a high degree of similarity with other already isolated HQT sequences [25,41] and with HCTs from globe artichoke [32], coffee [41], tobacco and Arabidopsis [24] (Fig. 3).

### Heterologous expression of globe artichoke HQT in *E. coli *and enzyme assays

**Figure 4:**
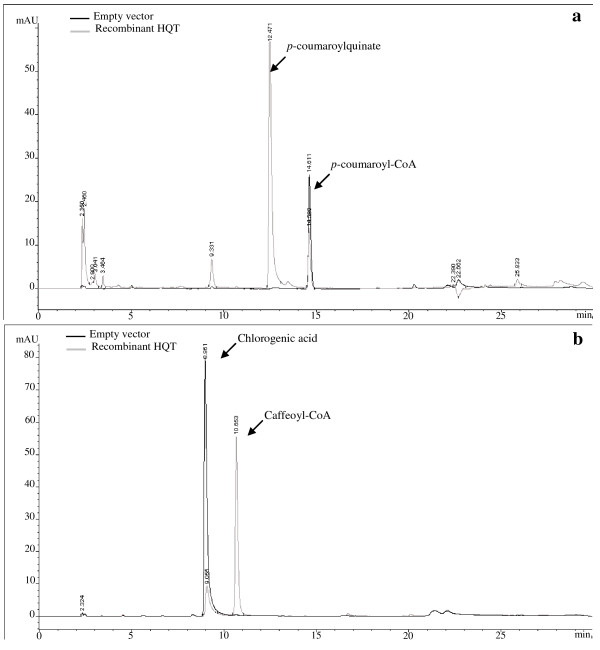
**HPLC analysis of the HQT reaction products**. An aliquot of the incubation reaction without (black line) or with (gray line) recombinant HQT was analysed. (a) HQT reaction with *p*-coumaroyl-CoA and quinate; (b) HQT reverse reaction with chlorogenic acid and CoA.

The globe artichoke acyltransferase cDNA was cloned and expressed in *E. coli*, using a pET3a expression vector. SDS-PAGE analysis demonstrated the presence of a heterologous protein of apparent molecular mass ~50 kDa (consistent with the predicted size of the transgene translation product) both in the supernatant and in the pellet fraction. This protein was absent from the equivalent fractions of cultures of bacteria carrying an empty pET3A plasmid. The recombinant enzyme was incubated in the presence of *p-*coumaroyl-CoA or caffeoyl-CoA and quinic acid or shikimic acid as substrates, and the products of the reactions were analyzed by HPLC. In the presence of active enzyme, new products (*p-*coumaroylquinate and caffeoylquinate) were detected in the reaction mixtures containing *p-*coumaroyl-CoA or caffeoyl-CoA and quinic acid. These products could not be detected when reactions were performed with the control crude extract (Fig. 4). No significant peaks were detected after addition of shikimic acid instead of quinic acid in the reaction mixture.

Each reaction product was identified by comparing its retention time and absorbance spectrum with authentic samples or isolated compounds previously characterized. The ability of the isolated acyltransferases to catalyse the reverse reaction (*i.e*. the production of caffeoyl-CoA from CGA) was also successfully achieved, as has been described in other systems [24,28,42]. Caffeoyl-CoA was detected when CGA was incubated with Coenzyme A in the presence of the recombinant protein (Fig. 4), whereas no metabolic product was detected from cultures carrying an empty plasmid.

### Real-time PCR

In order to assess the involvement in the response to UV-C irradiation, the expression levels of HQT and HCT were analysed by real-time PCR. Based on normalized levels (using actin as an internal standard), it was clear that UV-C treatment induced a significant increase in transcription (12.3 ± 1.8 fold for HCT and 4.4 ± 0.7 fold for HQT). Comparison between the standard curves for each enzyme revealed a correlation coefficient of > 0.98 and an efficiency (slope of the curve) > 0.90 (data not shown).

### Linkage analysis

**Figure 5:**
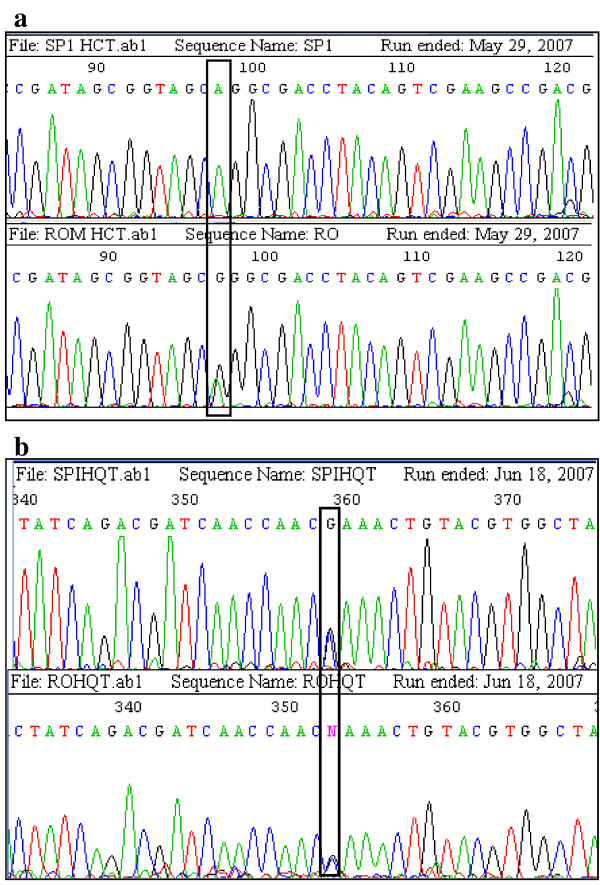
**SNP markers development**. Inter-varietal polymorphism between HCT (A) and HQT (B) genomic sequences of parental genotypes used for genetic mapping in globe artichoke [36]. The black frames showed SNPs used for designing primers employed in the tetra-primer ARMS PCR reactions.

**Figure 6:**
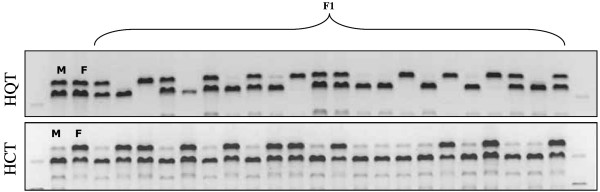
**Results of tetra-primer ARMS PCR reactions**. Segregation of HQTsnp359 (a) and HCTsnp97 (b) in the mapping population, as detected by tetra-primers ARMS-PCR on 2% agarose gel. M = male parent and F = female parent.

**Figure 7:**
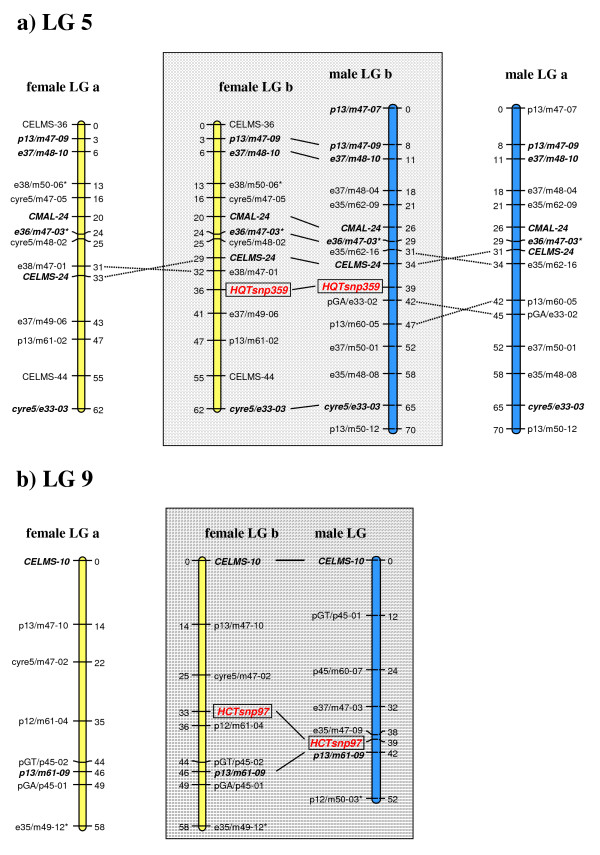
**Linkage groups (LG) 5 and 9 in globe artichoke maps**. LG5 (a) and LG9 (b) of the globe artichoke varietal types 'Romanesco C3' (female parent, yellow LGs on the left) and 'Spinoso di Palermo' (male parent, blue LGs on the right). LGs with HCT and HQT genes are reported in gray boxes, intercross markers are shown in bold and are connected by a solid line. The LGs previously reported by Lanteri *et al*. [36] are presented to one side, and changed marker orders are indicated by dotted lines. Asterisks indicate markers showing significant levels of segregation distortion (*: 0.1 > P ≥ 0.05, **: 0.05 > P ≥ 0.01).

Two single nucleotide polymorphisms (SNPs, HQTsnp359 and HCTsnp97) were identified (Fig. 5) in the HQT and HCT parental sequences. Both parents of the mapping population were heterozygous for marker HQT-snp359 (parental genotypes ab × ab), that segregated in the ratio 1:2:1 (aa:ab:bb) in the F_1 _individuals, with no evidence of any segregation distortion (Table 1, Fig. 6). This allowed the HQT gene to be placed on linkage group (LG) 5 in both the female and male maps (Fig. 7a). A further 14 markers were assigned to the female LG5: four microsatellites (CELMS-24, -36, -44 and CMAL-24), three S-SAPs (cyre5 markers) and 7 AFLPs, covering 62.1 cM and a mean inter-marker distance of 4.4 cM. More than 70% of intervals are < 4 cM in genetic length, with four gaps of > 6 cM. In addition to the HQT locus, the male LG5 consists of 15 markers: two SSRs (CELMS-24 and CMAL-24) one S-SAP, one M-AFLP (polyGA marker) and 11 AFLPs, spanning 69.5 cM and a mean inter-marker distance of 4.4 cM (range 1.6–7.7). Seven markers (including HQT-snp359) were shared between the parents, allowing the alignment of their LG5. The HQT locus maps close to AFLP markers e38/m47-01 and e47/m49-06 in the female map, and to the M-AFLP marker polyGA/e33-02 and the microsatellite CELMS-24 in the male map (Fig. 7a).

**Table 1:** Model, expected and observed segregation ratios of SNPs developed from HQT and HCT genes in the F_1 _progeny.

**Marker**	**Parental genotypes** **(Female × Male)**	**Expected ratios and F_1 _plant genotypes**	**Observed ratios**				**χ^2^**
				
			**aa**	**ab**	**bb**	**total**	
**HQTsnp359**	ab × ab	1: 2: 1 (aa: ab: bb)	21	44	28	93	1.32 ns
**HCTsnp97**	ab × aa	1: 1 (aa: ab)	50	43	-	93	0.53 ns

Only the female parent was heterozygous at HCTsnp97, delivering a segregation ratio of 1:1 with no significant distortion (Table 1, Fig. 6). As a result, the HCT gene could only be located on the maternal map, where it maps to LG9, separated by 3 cM from the AFLP locus p12/m61-04 and by 8 cM from the SSAP locus cyre5/m47-02 (Fig. 7b). A further six markers are present on this 58.4 cM LG, including one SSR (CELMS-10), two M-AFLPs (polyGT and polyGA) and three AFLPs. The marker density is 7.3 cM (range 1.6–7.7), with two gaps of > 10 cM.

## Discussion

Plants synthesize a variety of secondary metabolites, which function as UV protectants, phytoalexins, flower pigments, signalling molecules and building blocks for lignin. Some have significance in the area of human health, both as 'phytomedicines', which target specific health problems, and/or as 'nutraceuticals', which provide long term nutritional benefit [43]. Particular plant PPs have been associated with anti-oxidant, estrogen-like and vasodilatory activity, while others have proven anti-inflammatory and anti-cancer chemopreventive action [29,44-48]

CGA is the most widespread plant PP. Progress is being made in relation to the definition of its biosynthetic pathway, with the characterisation of two acyltransferases (HCT, [24] and HQT, [25]) able to synthesize *p-*coumaroylshikimate and *p-*coumaroyl quinate esters and a cytochrome P450 *p-*coumaroyl ester 3'-hydroxylase (C3'H) from a *p-*coumaroyl ester substrate [49,50].

The major phenolic compounds present in the leaves of the globe artichoke are the DCQs, and their precursor CGA. Although there is no firm proof as yet that DCQ originates from CGA, the structural similarity of the two molecules makes this rather likely. A globe artichoke acyltransferase involved in PP synthesis responded to both *p-*coumaroyl-CoA and caffeoyl-CoA esters as acyl donors [32].

In the present study, we have described *C. cardunculus *sequences carrying peptide motifs characteristic of the plant acyltransferase family. These sequences cluster within the N-hydroxycinnamoyl/benzoyltransferase group [51] and are closely related to their tobacco and tomato orthologues. The hydroxycinnamoyltransferase activity of the enzyme and its involvement in PP biosynthesis have been confirmed by heterologous expression assays, which showed that it can use either *p-*coumaroyl-CoA or caffeoyl-CoA esters as an acyl donor, and can use quinic acid as an acceptor. As the HQT gene product failed to utilize shikimic acid, we believe that it is involved in the transesterification of caffeoyl-CoA and quinic acid, a reaction which occurs in the first route of CGA biosynthesis, but also at the end of the third pathway, following the action of HCT and C3'H resulting in the formation caffeoyl-CoA.

PP metabolism can be induced by the application of abiotic stresses [21,52] and it has been shown that PP leaf content of globe artichoke mostly responds to UV-C irradiation, as compared to other treatments such as methyljasmonate and salicylate that are inactive [23]. Here, we have investigated the effect of UV-C irradiation on the transcription level of the HCT and HQT genes involved in the caffeoylquinic acid pathway. The transcription of both genes was induced by UV-C, suggesting their involvement in the higher production of PPs observed as the response to this stress. Previous work on globe artichoke demonstrated that UV-C application led to large increases of leaf DCQs whereas no significant effect was observed on CGA [23]. On the basis of our data this might be a consequence of the rapid conversion of CGA to DCQs as by means of an unknown downstream enzymatic step. Indeed the involvement of the HQT gene in the profile of phenolic acids accumulated can influence the kind of response to the UV stress as reported in a previous study on tomato by Clè *et al*. [20].

The genetic mapping of biosynthetic pathway genes of known biochemical function can help unravel the complexity of plant secondary metabolism. The precision of both marker order and inter-marker distances on LG5 and LG9 have been improved with the integration of the HQT and HCT genes. The former increased the number of bridge markers on LG5, and reduced some large gaps (of 10 cM and 8 cM) affecting the female and the male LGs (Fig. 7a). Its incorporation has caused some readjustment in the marker orders and inter-marker distances determined previously [36]. Thus in the female LG, the order of CELMS24 and e38/m47-01 was inverted, as were those of CELMS-24, e35/m62-16 and pGA/e33-02, p13/m60-05 on the male LG. The placement of the HCT gene on female LG9 did not increase the number of bridge markers, nor did it affect marker order. However, it did succeed in filling a large (13 cM) gap, and in reducing the mean inter-marker distance. Increasing marker density by the addition of genes to a map can be accomplished via the exploitation of mapping populations which segregate for traits and markers in common across the populations [53,54]. We are currently constructing further genetic maps based on combinations between 'Romanesco C3' and either cultivated or wild cardoon accessions, primarily as a means of initiating comparative QTL mapping. Within gene markers, such as the ones described here for the HCT and HQT genes, are particularly suitable for general mapping, and should prove useful as anchor points among diverse populations.

## Conclusion

A novel acyltransferase involved in the biosynthesis of CGA in globe artichoke has been isolated and characterized. Its activity and involvement in CGA biosynthesis have been confirmed by heterologous expression assays, demonstrating that it can use either *p-*coumaroyl-CoA or caffeoyl-CoA as an acyl donor, and quinic acid as an acceptor. We previously observed that the PP metabolism can be induced by UV-C irradiation, whose effect on the transcription level of the HCT and HQT genes has been investigated. The HQT as well as HCT genes have been located in our previously developed globe artichoke genetic maps; the linkage analyses of genes having known biochemical function can help elucidate the complexity of plant secondary metabolism.

This work is a further contribution in the understanding of the genetic basis of phenylpropanoid (PP) biosynthesis in *C. cardunculus*; our future research activity will be focused on the analysis of the expression *in vivo *of both HQT and HCT, as well as on isolating further acyltransferases involved in the phenylpropanoid pathway of the species.

## Methods

### Plant material and RNA extraction

Leaves of globe artichoke, and cultivated cardoon were collected from experimental fields in Scalenghe, Torino (Italy).

Total RNA was extracted from approximately 100 mg fresh tissue using the "Trizol" reagent (Invitrogen, USA), following the manufacturer's instructions. Final RNA concentration was determined by spectrophotometry, and its integrity was assessed by electrophoresis in 1% (w/v) formaldehyde-agarose gel [55].

### Isolation and cloning of full length cDNA of globe artichoke and cardoon

Reverse transcription from both globe artichoke and cardoon total RNA was achieved using poly(dT)primer and M-MuLV RNaseH-RT (Finnzymes, Finland), following the manufacturer's instructions. The PCR amplification of cDNA sequences was performed as described in Comino *et al*. [32], using primers (Table 2) designed according to the CODEHOP strategy [37,56]. A first amplification step was performed using as primers CODhqtFor and CODhqtRev (Table 2), designed on conserved regions after alignment (Clustal W at ) of HQT amino acid sequences available in Genebank: *Nicotiana tabacum *(CAE46932) and *Lycopersicum esculentum *(CAE46933). Products were run on 1% agarose gel and fragments of expected size were isolated and sent to BMR genomics  for sequencing. To obtain the full length sequence, specific primers based on both, globe artichoke and cardoon, partial cDNA sequences, were designed for 3'- and 5'-end amplification as described in Comino *et al*., [32]. Using ClustalW with standard parameters, the *C. cardunculus *full length amino acid sequences were aligned with the publicly available acyltransferases transferring hydroxycinnamoyl groups to acceptors from the shikimate pathway. Phylogenetic analysis was conducted using MEGA version 3.0 [57].

**Table 2:** Oligonucleotide sequences used to study HQT gene in *C. cardunculus*.

**Primer**	**Sequence (5'-3')**
**CODhqtFor**	AAGCCNWSNAARCC
**CODhqtRev**	CCCCANCCRAARTC
**HQT-For**	GGGTTTCATATGACTATCGGAGCTCGTGAT
**HQT-Rev**	CGGGATCCCTAGAAGTCATACAAGCATTT
**HCT-ForRT**	TTTTTAAGCTAACACGAGAC
**HCT-RevRT**	TCTCATAGGAGCTGTAATTG
**HQT-ForRT**	TAAAATGGACGATCAGTATC
**HQT-RevRT**	TTATGTTCAGATTTGGACTC
**ACT-ForRT**	TACTTTCTACAACGAGCTTC
**ACT-RevRT**	ACATGATTTGAGTCATCTTC
**HCT-For**	GGGTTTCATATGAAGATCGAGGTGAGAGAA
**HCT-Rev**	CGGGATCCTTAGATATCATATAGGAACTTGC
**HCT-InnerFor**	ATATTCACGACGACTCCGATAGCGGTATCG
**HCT-InnerRev**	CACGTCGGCTTCGACTGTAGGTCGACT
**HCT-OuterFor**	CACGAGACCAAGTCAATGCACTCAAAGGA
**HCT-OuterRev**	GATTCGGGCACTTAAACGTATGAGCCCC
**HQT-InnerFor**	CGTGGACTATCAGACGATCAACCATCC
**HQT-InnerRev**	TCGTCCGTCAGTAGCCACGTACAGTATC
**HQT-OuterFor**	CACAAAACCAAAACTTCACATCCCATCC
**HQT-OuterRev**	CTCACTATGGATTCTCCTAGCGGTGTCG

### Heterologous expression of globe artichoke HQT in *E. coli *and enzymatic assays

The globe artichoke HQT open reading frame (ORF) was amplified using HQT-For and HQT-Rev primers (Table 2), which contain additional restriction sites, respectively, *Nde*I (5'-end) and *Bam*HI (3'-end). In a first step the amplified fragment was digested with *Nde*I and partially with *Bam*HI (15 min at 37°C with 1 unit of *Bam*HI). This partial second digestion being necessary because of the presence of an internal *Bam*HI restriction site. The restricted PCR fragment was finally ligated into the cloning site of *Nde *I – *Bam *HI digested pET3a plasmid (Novagen, USA). The resulting recombinant pET3a-HQT plasmid was transferred into *E. coli *strain BL21(DE)pLysE, and grown on a selective medium (LB in presence of 50 mg/l ampicillin and 34 mg/l chloramphenicol). Individual colonies were transferred to 4 ml LB medium and incubated for 12 h at 37°C. Two ml of this bacterial preculture were transferred in 50 ml LB medium and grown for 3 h at 28°C prior to an isopropyl-β-D-thiogalactopyranoside (IPTG) induction (final concentration of 1 mM) during 8 h at 28°C. After centrifugation for 10 min at 5000 g, the pellet was resuspended in 1 ml of phosphate-buffered saline pH 7.5 and lysed by three cycles of freezing (in liquid nitrogen) and thawing (at 37°C), followed by three bursts of 30 s sonication on ice. Sonicated cells were centrifuged at 4°C and 14,000 g for 5 min, and the supernatant was assayed for HQT activity, and profiled by SDS-PAGE (10% resolving gel, 5% stacking gel) using Coomassie brilliant blue staining [55]. Negative controls used comparable preparations harbouring an empty vector.

The recombinant proteins were used for enzyme assays. CGA was purchased from Sigma-Aldrich (Germany), and quinic acid from Fluka (Switzerland). CoA esters (substrates) were synthesised using the procedure proposed by Beuerle and Pichersky [58]. 4CL enzyme was kindly provided by Dr. Douglas (University of British Columbia, Vancouver).

The 20 μl reaction mixture contained 100 mM phosphate buffer (pH 7.5), 1 mM dithiothreitol, between 50 ng and 1 μg of protein, and the various substrates (*p*-coumaroyl-CoA, caffeoyl-CoA, quinic acid and shikimic acid) at concentrations ranging from 0.1 mM to 5 mM. The reverse reaction, i.e. conversion of chlorogenic acid and CoA-SH (Sigma) into caffeoyl-CoA, was tested as follow: 50 ng to 1 μg protein was incubated in presence of 1 mM dithiothreitol, 100 μM of chlorogenic acid and 100 μM CoA. Reactions were incubated at 30°C for 30 min, stopped by the addition of 20 μl of acetonitrile/HCl (99:1) and products were analysed by reverse-phase HPLC on a C18 column (LiChroCART 125-4, Merck). The two solvents used are 90% H_2_O, 9.9% CH_3_CN, 0.1% HCOOH and 80% CH_3_CN, 19.9% H_2_O, 0.1% CH_3_COOH. The percentage of the latter reached the 60% over a 15 min run time, and 100% after 28 min.

### Real-time PCR experiments

For real-time PCR assays, UV-C stress experiments are performed as described in Moglia *et al*., [23]. Total RNA was extracted as described above. The first-strand cDNA was synthesised using iScript cDNA Synthesis Kit (Biorad), following manufacturer's instructions.

Primers (HCT-ForRT, HCT-RevRT, HQT-ForRT, HQT-RevRT, Table 2) were designed on HCT (DQ104740), and HQT (DQ915589) sequences using the Primer 3 software [59]. As a housekeeping gene, actin was chosen for its stability and level of expression, which is comparable to the genes of interest and whose expression remained stable after the UV-C stress. The primers (ACT-ForRT, ACT-RevRT, Table 2) for its amplification were designed on the artichoke actin (ACT, AM744951). All primers were purchased from Metabion (Germany).

Standard curves were prepared for both the housekeeping ACT and target genes. The cDNAs were performed in triplicate for each sample in 20 μl. Reaction mixes contained 2× iQ SYBR Green Supermix (Bio-Rad Laboratories, USA), specific primers at 300 nM, and 3 μl of cDNA. PCR reactions were carried out in 48-well optical plates using the iCycler Real-time PCR Detection System (Bio-Rad Laboratories, USA). Cycling parameters were as follows: one cycle at 95°C for 5 min for DNA polymerase activation, followed by 35 cycles of 5 sec at 95°C (denaturation) and 20 sec at 60°C (annealing and extension). In all experiments, appropriate negative controls containing no template were subjected to the same procedure to exclude or detect any possible contamination. Melting curve analysis was performed at the end of amplification. Standard curves were analyzed with the iCycler iQ software. This quantification system was designed to automate analysis options, including quantitative and melting curve analysis. The results of amplification were analyzed by the comparative threshold cycle method, also known as the 2-ΔΔCt method [60]. This method compares, for each time-point considered, the Ct values of the samples of interest (CtI) with the appropriate calibrator (CtM). The Ct values of both the calibrator and the samples of interest are normalized to the housekeeping gene.

### SNP detection and linkage analysis

The allelic forms of globe artichoke HCT (isolated in the previous work) [32] and HQT (this work) were analysed in the two globe artichoke genotypes ('Romanesco C3' and 'Spinoso di Palermo'), previously used for map development [36]. The full length HCT and HQT sequences were amplified on parental genome with 2 sets of primers (one for each isolated gene, HCT-For, HCT-Rev, HQT-For and HQT-Rev reported in Table 2) and PCR products were sequenced for SNP identification. SNPs genotyping were carried out with the tetra-primers ARMS-PCR method [61,62] by using two sets of outer and inner primers (Table 2), designed using the software made available on line . PCR products were separated by 2% agarose gel electrophoresis.

Segregation data of HCT- and HQT-SNP markers were monitored and analyzed together with those of AFLP, S-SAP, M-AFLP and SSR markers previously applied for globe artichoke maps construction [36]. The goodness-of fit between observed and expected segregation data was assessed using the chi-square (χ^2^) test. Independent linkage maps were constructed for each parent using the two way-pseudo testcross mapping strategy [63] by using JoinMap 2.0 software [64]. For both maps, linkage groups were accepted at a LOD threshold of 4.0. To determine marker order within a linkage group, the following JoinMap parameter settings were used: Rec = 0.40, LOD = 1.0, Jump = 5. Map distances were converted to centiMorgans using the Kosambi mapping function [65]. Linkage groups were drawn using MapChart 2.1 software [66].

## Authors' contributions

SL and FB planned and supervised the work. CC, AM, BM and AH carried out the molecular genetic studies; EP carried out phylogenetic and linkage analyses. All authors read and approved the final manuscript.
